# Rate Sensitive Continuum Damage Models and Mesh Dependence in Finite Element Analyses

**DOI:** 10.1155/2014/260571

**Published:** 2014-11-03

**Authors:** Goran Ljustina, Martin Fagerström, Ragnar Larsson

**Affiliations:** Division of Material and Computational Mechanics, Department of Applied Mechanics, Chalmers University of Technology, 412 96 Gothenburg, Sweden

## Abstract

The experiences from orthogonal machining simulations show that the Johnson-Cook (JC) dynamic failure model exhibits significant element size dependence. Such mesh dependence is a direct consequence of the utilization of local damage models. The current contribution is an investigation of the extent of the possible pathological mesh dependence. A comparison of the resulting JC model behavior combined with two types of damage evolution is considered. The first damage model is the JC dynamic failure model, where the development of the “damage” does not affect the response until the critical state is reached. The second one is a continuum damage model, where the damage variable is affecting the material response continuously during the deformation. Both the plasticity and the damage models are rate dependent, and the damage evolutions for both models are defined as a postprocessing of the effective stress response. The investigation is conducted for a series of 2D shear tests utilizing different FE representations of the plane strain plate with pearlite material properties. The results show for both damage models, using realistic pearlite material parameters, that similar extent of the mesh dependence is obtained and that the possible viscous regularization effects are absent in the current investigation.

## 1. Introduction

The traditional Johnson-Cook (JC) plasticity model [1] is often used in finite element (FE) analysis of metal cutting. From the industrial perspective, one important advantage of the JC model, compared to many others, is its availability as built in constitutive model in the commercial software packages. In the JC model, it is assumed that the flow stress is a unique function of the total strain, plastic strain rate, and temperature and that their effects on the flow stress can be described in a multiplicative fashion.

The accuracy of phenomenological models, like the JC plasticity model, is often satisfactory in the range of deformation conditions for which they were curve fitted. What is missing is the ability to capture the kinematic hardening, recovery, or complex loading mechanisms that are common in machining. On the other hand, as one example of dislocation mechanics based models, the BCJ (Bammann-Chiesa-Johnson) model [2, 3] has been developed to incorporate the loading effect in complex material deformations and is successfully used earlier in orthogonal machining simulations of the cast iron material (cf. [4, 5]).

Machining simulation models in many cases include damage models in order to describe the development of segmented or discontinuous chips (cf. [6–11]). In the earlier contributions [12, 13], a Johnson-Cook (JC) plasticity model was used along with the Johnson-Cook dynamic failure criterion to describe deformation and damage in 2D orthogonal machining simulations with a FE-resolved compacted graphite iron (CGI) microstructure.

The JC dynamic failure [14, 15] defines a simple damage/failure model when the “damage” development in simulations is manifested through an accompanying element deletion procedure. The element is deleted when the accumulated plastic strain reaches a critical value, and there is thus no “damage” influence on the stress response before the critical plastic strain is reached and the element is deleted. This critical or failure strain is dependent on a nondimensional plastic strain rate, stress triaxiality, and temperature. Hence, due to the fact that the Johnson-Cook dynamic failure model is not affecting the stress response until the failure strain is reached, the model will be referred to as the “*uncoupled damage*” (UD) model from here on. Despite the fact that rate dependence is included in the Johnson-Cook plasticity model, and thereby a certain “viscous regularization” [16, 17] can be expected, our experience with machining simulations using this model indicates the presence of the pathological mesh dependence [18, 19]. Thereby, the aim of the present paper is to investigate the significance of the pathological mesh dependence obtained for different FE representations of the plane strain plate with pearlite material properties.

Another failure model that has been successfully used in machining simulations of cast iron with resolved microstructure in the literature (cf. [4]) is the damage model describing spherical voids growth by Cocks and Ashby [20]. In contrast to the JC dynamic failure model, the effective stress response is in this model reduced by a scalar damage variable in the plastic deformation phase creating a more realistic stress-strain pattern. Unlike the uncoupled damage model that requires five material parameters (*d*_1_–*d*_5_), one single material parameter is needed for this model. This damage model is referred to as the “*continuum damage*” (CD) model in this paper. So, the additional objective of this contribution is to compare the two damage models with respect to pathological mesh dependence.

## 2. Damage Models Based on Viscoplastic Evolution

In this section we describe two fundamentally different damage/failure models, used to represent ductile fracture of the pearlite material. In the continuum damage model, the evolving damage is affecting the response progressively as the plastic deformation develops, whereas in the uncoupled damage model the evolved damage takes place in one single step when the plastic strain reaches a critical value. Both models rely on the Johnson-Cook model for the representation of the effective stress response.

### 2.1. Effective Material and the Johnson-Cook Model

It is assumed that the Helmholtz free energy $\bar{\psi }$ of the effective (undamaged) material, denoted by a superimposed bar, has the dependencies

(1) $$\begin{array}{c} \bar{\psi }=\bar{\psi }\left[C,k\right], \end{array}$$

where **C** = **F** ^*t*^ · **F** (where **F** is the deformation gradient) is the elastic right Cauchy-Green deformation tensor and *k* is the isotropic hardening variable. It then appears that the dissipation rate $\bar{D}$ of the dissipation inequality becomes

(2) $$\begin{array}{c} \bar{D}=\bar{\tau }:l_{p}+\bar{\kappa }\dot{k}\geq 0 \end{array}$$

corresponding to the constitutive state equations

(3) $$\begin{array}{c} \bar{S}=\frac{\partial \bar{\psi }}{\partial \bar{C}}⟹\bar{\tau }=F\cdot \bar{S}\cdot F^{t},\;\;\bar{\kappa }=-\frac{\partial \bar{\psi }}{\partial k}. \end{array}$$

In (3), we introduced the effective Kirchhoff stress $\bar{\tau }$ related to the effective second Piola Kirchhoff stress $\bar{S}$ with the usual transformation. Moreover, $\bar{\kappa }$ is the hardening stress pertinent to the effective material. In view of (2), let us introduce the evolution of the internal dissipative variables $\left\{l_{p},\dot{k}\right\}$ in terms of the yield function of the effective stress; that is, $\Phi =\Phi [\bar{\tau },\bar{\kappa }]$. The evolution of the internal variables is thus formulated as

(4) $$\begin{array}{c} l_{p}=\lambda \frac{\partial \Phi }{\partial \bar{\tau }}=\lambda f⟹f=\frac{\partial \Phi }{\partial \bar{\tau }}=\frac{3}{2}\frac{{\bar{\tau }}_{\text{dev}}}{{\bar{\tau }}_{e}},\;\;\dot{k}=\lambda \frac{\partial \Phi }{\partial \bar{\kappa }}, \end{array}$$

where *λ* ≥ 0 is the plastic multiplier (pertinent to the effective inelastic response) and **f** is the gradient of the yield function in terms of the* effective* Kirchhoff stress $\bar{\tau }$. As alluded to in the yield function $\Phi =\Phi [\bar{\tau }]$, von Mises plasticity is considered involving the von Mises stress ${\bar{\tau }}_{e}$ defined by

(5) $$\begin{array}{c} {\bar{\tau }}_{e}=\sqrt{\frac{3}{2}}\left|{\bar{\tau }}_{\text{dev}}\right|. \end{array}$$

As to the plastic multiplier *λ* ≥ 0, let us consider the* Johnson-Cook* model [1] where the overstress function is specified in the quasi-static yield function as

(6) $$\begin{array}{c} \lambda =\left\{\begin{array}{cc} {\dot{\epsilon }}_{0}\exp\left[\frac{\Phi }{C\left(1-{\hat{\theta }}^{m}\right)\left(A+Bk^{n}\right)}\right] & \frac{\lambda }{{\dot{\epsilon }}_{0}}\geq 1\\ \Phi \leq 0,\;\;\lambda \geq 0,\;\;\lambda \Phi =0 & \frac{\lambda }{{\dot{\epsilon }}_{0}}<1, \end{array}\right. \end{array}$$

where *k* = *ϵ*_*e*_^*p*^ is the internal hardening variable (equal to the equivalent effective plastic strain) and $\lambda =\dot{k}={\dot{\epsilon }}_{e}^{p}$. Furthermore, the quasi-static yield function is defined as

(7) $$\begin{array}{c} \Phi ={\bar{\tau }}_{e}-\left(A+B{\left(k\right)}^{n}\right)\left(1-{\hat{\theta }}^{m}\right). \end{array}$$

We conclude that the response becomes rate dependent (or viscoplastic) whenever $\lambda \geq {\dot{\epsilon }}_{0}$. The response becomes rate independent whenever $\lambda <{\dot{\epsilon }}_{0}$, controlled by usual loading conditions introduced in (6). Moreover, the factor $\left(1-{\hat{\theta }}^{m}\right)$ involves the temperature dependence where $\hat{\theta }$ is the homologous temperature and *m* is a parameter (cf. [1, 13]). As to the material parameters involved, we note that *A*, *B*, and *C* are material parameters representing initial yield, hardening, and rate sensitivity, respectively. In addition, the exponent *n* represents the hardening. Concerning the parameter ${\dot{\epsilon }}_{0}$, we note that it has a strong influence on the rate sensitivity.

### 2.2. A Damage Enhanced Formulation

In the present context it is of significant interest to enhance the effective material in terms of a scalar (isotropic) damage measure *ϕ* acting on the effective material so that

(8) $$\begin{array}{c} \psi =\left(1-\phi \right)\bar{\psi } \end{array}$$

whereby the total dissipation rate *D* becomes extended as

(9) $$\begin{array}{c} D=\left(1-\phi \right)\bar{D}+\bar{\psi }\dot{\phi }\geq 0, \end{array}$$

where (again) $\bar{D}$ is the effective dissipation rate. To ensure positive dissipation *D* ≥ 0 it suffices to consider $\bar{D}\geq 0$ as in the sequel (2)–(4) and $\dot{\phi }\geq 0$ (since $\bar{\psi }\geq 0$ always). This corresponds to the constitutive state equations:

(10) $$\begin{array}{c} \tau =\left(1-\phi \right)\bar{\tau },\;\;\kappa =\left(1-\phi \right)\bar{\kappa },\\ A=-\frac{\partial \psi }{\partial \phi }=\bar{\psi }. \end{array}$$

We thus conclude that, for example, the total Kirchhoff stress is obtained via the relation $\tau =(1-\phi )\bar{\tau }$ and that *A* is the damage force driving the damage evolution via the elastically stored effective energy $\bar{\psi }$.

### 2.3. A Continuum Damage Model

The structure proposed in the previous subsection involves a completely decoupled formulation of the effective stress response from damage. As alluded to in the sequel (8)–(10) the damage evolution is defined as a postprocessing of the effective stress response. Concerning the damage evolution $\dot{\phi }\geq 0$, we consider developments in, for example, Chuzhoy et al. [4] and Cocks and Ashby [20] and propose

(11) $$\begin{array}{c} \dot{\phi }=\left(1-\phi \right)\left({\left(1-\phi \right)}^{-\left(n_{d}+1\right)}-\left(1-\delta \right)\right)\beta \left[r\right]\lambda \geq 0, \end{array}$$

where we introduced a slight adjustment to its original expression in terms of the parameter *δ* (normally set to *δ* = 1%), is introduced in order to define the effect of materials integrity in the beginning of the damage process. Furthermore, *n*_*d*_ is the damage exponent parameter for the material.

Another adjustment of the original damage evolution law refers to the *β* function which is the stress triaxiality factor defined by

(12) $$\begin{array}{c} \beta =\left\{\begin{array}{cc} \sinh\left[\frac{2\left(2n_{d}-1\right)}{2n_{d}+1}r\right] & r\geq 0.8\\ k_{1}\exp\left[k_{2}r\right] & r<0.8, \end{array}\right. \end{array}$$

where $r=-\bar{p}/{\bar{\tau }}_{e}$ and $\bar{p}=-(1/3)1:\bar{\tau }=-(1/3)1:{\bar{\tau }}^{\mathrm{tr}}$. An enhancement of the original *β*-function in (12) is made to avoid unphysical negative *β*-values. This occurs for triaxiality ratios *r* ≤ 0.8, where a new exponential branch to the hyperbolic function is defined. The parameters *k*_1_ and *k*_2_ are determined at the point *r* = 0.8 so that

(13) $$\begin{array}{c} \sinh\left[\frac{2\left(2n_{d}-1\right)}{2n_{d}+1}r\right]=k_{1}\exp\left[k_{2}r\right],\\ \sinh{\left[\frac{2\left(2n_{d}-1\right)}{2n_{d}+1}r\right]}^{\prime }=k_{1}k_{2}\exp\left[k_{2}r\right]. \end{array}$$

### 2.4. An Uncoupled Damage Model

Parallel to the continuum damage model outlined in the previous subsection we shall consider the uncoupled damage model of Johnson and Cook [14]. A “damage” measure *D*, represented by the accumulated plastic deformation at the “current” time *t*, is introduced as

(14) $$\begin{array}{c} D=\overset{t}{\underset{0}{\int }}\frac{\lambda }{\epsilon_{f}^{p}\left[t\right]}dt. \end{array}$$

Whenever *D* reaches the value 1 at any one integration point in an element the element is removed from the mesh following the procedure of the element removal technique used in the analysis. For the review of the element removal method compare, for example, [21]. According to the Johnson-Cook model, the fracture strain is expressed by three dependencies in a multiplicative fashion (like in the JC material model). The dependencies are those of stress triaxiality, strain rate, and temperature formulated in terms of the equivalent plastic fracture strain *ϵ*_*f*_^*p*^ defined as

(15) $$\begin{array}{c} \epsilon_{f}^{p}=\left(d_{1}+d_{2}\exp\left[-d_{3}r\right]\right)\left(1+d_{4}\ln\left[\frac{\lambda }{{\dot{\epsilon }}_{0}}\right]\right)\left(1+d_{5}\hat{\theta }\right). \end{array}$$

The presence of hydrostatic tension significantly decreases the level of critical plastic strain at which the material is considered to fracture. This is because nucleation, growth and coalescence of voids (being the major driving force of ductile fracture) are generally promoted by the hydrostatic tensile stress. The five material parameters in the failure criterion are the initial failure strain *d*_1_, the exponential factor *d*_2_, the triaxiality factor *d*_3_, the strain rate factor *d*_4_, and the temperature factor *d*_5_. Although the influence on the stress response is different for the uncoupled and continuum damage models, the development of the “damage” variable is controlled by the triaxiality ratio *r*, the strain rate, and the temperature in both cases. We note that the same dependencies in both failure models open up for the possibility to fit the associated damage parameters to obtain a calibrated similar response from both damage models.

## 3. Mesh Dependence Investigation

It is well known that local damage models generally lead to a pathological mesh dependence in the FE representation of localized plastic deformation. It is, however, argued in the literature (cf., e.g., [17]) that a viscous regularization of the continuum material model, for example, via viscoplasticity, may act as a localization limiter. To investigate the significance of this statement, the two (rate dependent) damage representations, described in the previous section, will be considered in the mesh dependency investigation.

The modeling of the effective material stress response (that serves as a basis for both the uncoupled and the continuum damage calculations) is based on the hypoelastic inelastic framework applied to the JC plasticity model [1]. Although the models are phrased in the thermodynamically consistent hyperelasticity framework, the hypoelastic inelastic framework is chosen due to its computational efficiency, as discussed in [13]. The hypoelastic inelastic response is postulated as

(16) $$\begin{array}{c} \hat{\bar{\tau }}=E_{e}:l_{e}\;\;\text{with}\;\;E_{e}=2GI_{\text{dev}}^{\text{sym}}+K1⊗1, \end{array}$$

where $\hat{\bar{\tau }}$ is the effective Green-Naghdi stress rate.

### 3.1. Matching the Parameters between Uncoupled Damage and Continuum Damage Formulations

**Figure 1:**
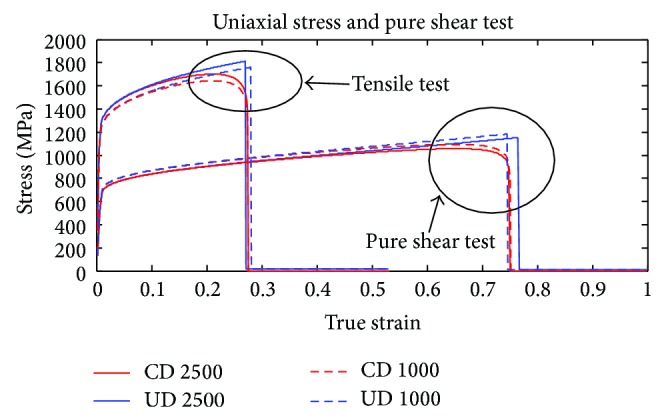
Resulting stress-strain behavior after the calibration of the CD and UD models based on uniaxial stress and pure shear deformation test with the applied loading rates 1000 and 2500 s^−1^.

In the previous subsection we outlined two fundamentally different damage/failure models. In the first coupled one, the damage is evolving progressively with the plastic deformation, whereas in the uncoupled one the evolved damage is assumed to take place in a single step, as discussed in Section 2.4. However, we note that the total stress response of the two models is comparable for fairly high values of the damage parameter (*n*_*d*_), corresponding to rapid damage evolution when *ϕ* → 1. It appears that the value *n*_*d*_ = 16 is reasonable from both experimental and numerical investigations of pearlitic steel at room temperature (cf. [5]). In this case, the parameters of the JC-failure model are calibrated to the parameters of the continuum damage model. To this end, the parameters* d*_1_–*d*_4_ of the uncoupled model are determined via least squares fitting from four loading cases. For this purpose, the uniaxial stress and simple shear tests at the* material point level* were considered with two different loading rates. The resulting stress-strain behavior after the calibration is shown in Figure 1, for each loading case and damage model. The nice fit between the models is noteworthy. The resulting parameters (*d*_1_–*d*_4_) are shown in Table 3.

The values of Young's modulus, Poisson's ratio, and density for the pearlite used in the simulations have been taken from the literature (cf. [4, 22] and cf. also Table 1 for representative values). The pearlite material parameters for the JC plasticity model (Table 2) are taken from [13] where the parameters are calibrated based on experimental data for pearlitic rail steels reported in [23, 24].

### 3.2. FE Analyses of a Shear Loaded Pearlite Plate

The mesh dependency investigation is performed both for the continuum damage model and for the uncoupled damage model. These two models have been used along with the hypoelastic inelastic framework applied to the JC-plasticity model and they have, for this purpose, been implemented in the commercial software Abaqus/Explicit as separate user subroutines.

The investigation is based on the results from the simulation of a shear loaded 2D-plate with (discussed earlier) pearlite material properties. The FE representations of the plate created are square shaped with dimensions of 50 × 50 mm. Different displacement rates were used (500, 2500, and 10000 mm/s) in the analyses. The calculations are performed isothermally in order to focus on the damage effect.

**Figure 2:**
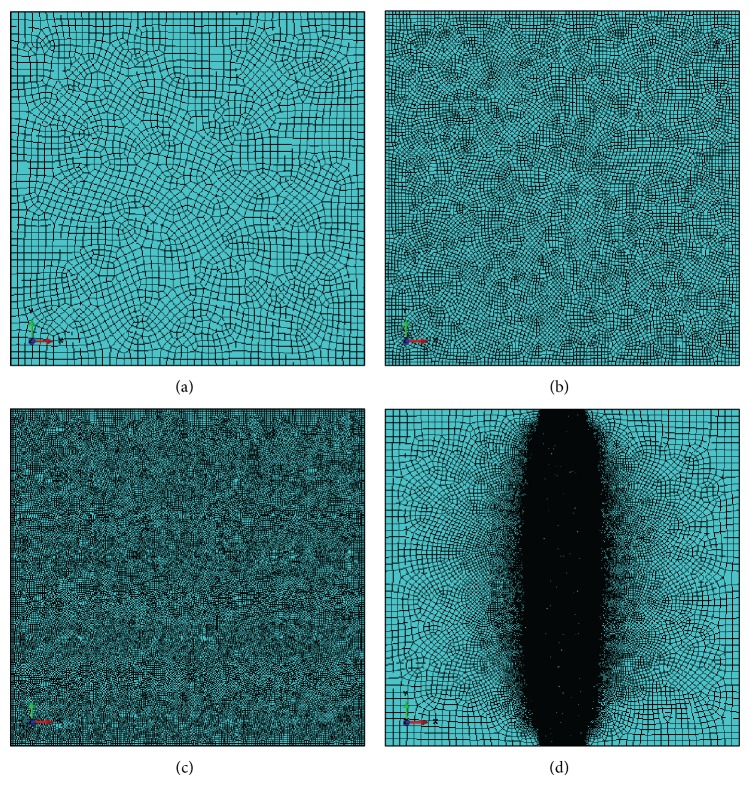
The considered FE representations of the plate based on different element sizes (1.0, 0.5, 0.3, and 0.1 mm corresponding to (a), (b), (c), and (d)). The meshes (a)–(c) are referred to as “coarse,” “mean,” and “fine,” whereas mesh (d) is referred to “extra fine.”

To be able to perform the mesh dependency investigation, four FE representations of the plate are created with four different sizes of the four-node plain strain bilinear elements and with approximate element sizes of 0.1, 0.3, 0.5, and 1.0 mm; the models contain 69921, 33899, 11933, and 2931 elements, respectively. The different FE representations of the plate are presented in Figure 2. The meshes (a)–(c) have uniform element size distributions, whereas mesh (d) of the plate has a finer element size in the mid area. Free distribution of the element nodes is used during creation of the meshes to avoid possible effects of structured mesh patterns.

**Figure 3:**
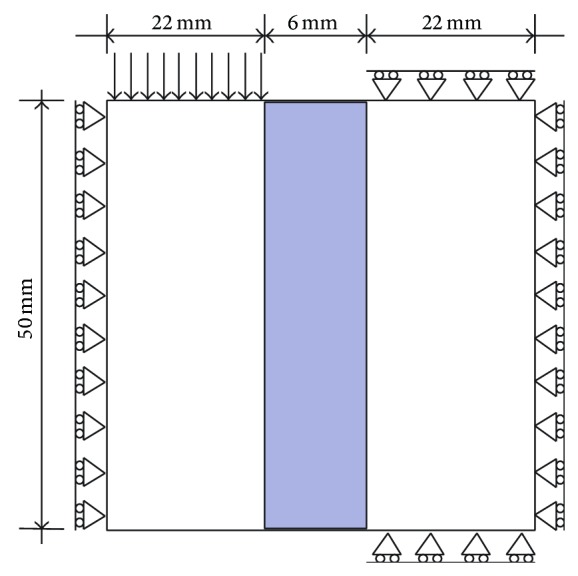
Considered shear loaded plate in plane strain.

The set-up for the shear test is shown in Figure 3. The mid area of the plane strain plate (highlighted in Figure 3) is exposed to severe shear deformation by prescribed vertical displacement (12.5 mm) downwards of the nodes on the left side of the top edge of the model. The nodes on the other side (right) of the localization area, lying both on the top and bottom edge, are constrained in the vertical direction. Also, the nodes on the left and the right plate edges are constrained in the horizontal direction.

## 4. Results

In this section, the simulation results in terms of shear deformations of different FE representations of the plate are compared and analyzed with respect to mesh dependence. Simulations are conducted based on both the uncoupled and the continuum damage models, with different displacement rates in order to reveal possible convergence with respect to mesh dependency due to viscous regularization. The study is conducted based on results in the form of the force-displacement curves, where the magnitude of the force is calculated at the edge where the displacement boundary conditions are defined.

We emphasize that the variation of the crack patterns and deformations obtained for different cases, where the involved damage models, displacements rates, and FE representations are varied, must also be taken into account when analyzing the results. In the simulations, where all four FE representations are used, the cracks are developing through one element row irrespectively of element size, which is typical for a pathological mesh dependency behavior. The overall impression is that simulations based on the uncoupled damage model generally behave more consistently regarding deformations and crack patterns obtained with different FE representations and displacement rates.

**Figure 4:**
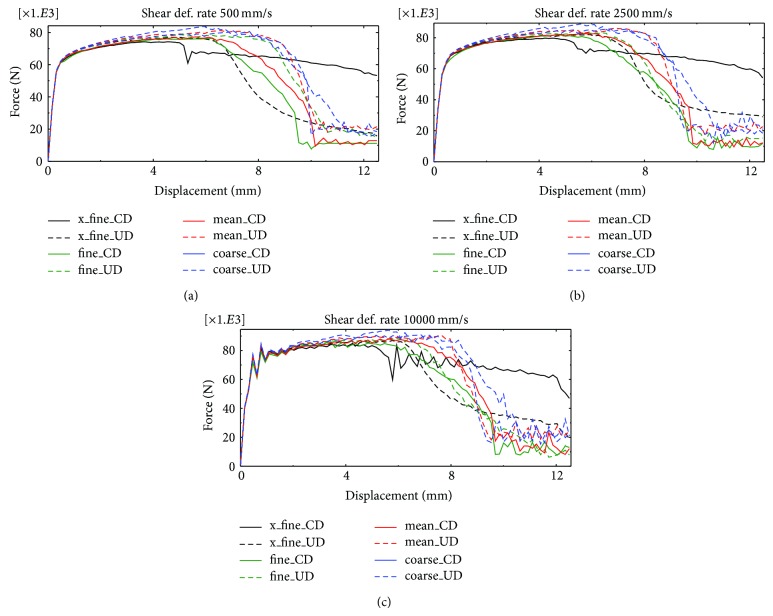
Force [N]-displacement [mm] curves from shear test analyses with displacement rates: (a) 500 mm/s, (b) 2500 mm/s, and (c) 10000 mm/s.

**Figure 5:**
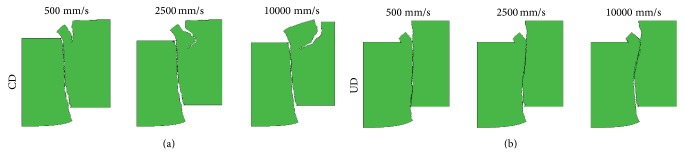
Variation of crack patterns in fine mesh for different displacement rates for (a) continuum damage model (CD) and (b) uncoupled damage model (UD).

The results of the simulations, in form of force-displacement curves, where both damage models and all element sizes were used, are plotted together in Figures 4(a), 4(b), and 4(c). As can be observed from the results, the damage development for the two damage models occurs almost simultaneously. This shows that the uncoupled and continuum damage models behave in a similar fashion in the shear test simulations, after the calibration of parameters of the uncoupled damage against the continuum damage model. If a comparison is made between the force-displacement results for different FE representations, a clear mesh dependence appears with a typical reduction of dissipated energy upon mesh refinement. This is despite the fact that a rate dependent model is utilized. This is illustrated, for instance, in the case of the continuum damage model (continuous lines) with displacement rate 2500 mm/s shown in Figure 4(b). In the case of the uncoupled damage model, the trend is the same until later in the damage development process where the curves (dashed lines) are crossing each other and the opposite trend is obtained which leads to smaller differences in energy dissipation compared to results based on the continuum damage model. If the crack patterns for the different displacement rates are compared, the only significant variation observed is in the case of the fine mesh (Figure 2(c)) for the simulations with the continuum damage model. Here, the location of the initiation point of the crack on the upper right edge of the plate moves to the right as the displacement rate is increased (cf Figure 5(a)).

A possible regularizing effect of higher deformation rates on the mesh dependency is also investigated by comparison of the inherent distances between force-displacement curves belonging to different displacement rates. Only a minor difference is observed and it was not possible to draw any general conclusions. As expected, in the simulations with displacement rate 10,000 mm/s the curves are becoming more uneven (due to dynamical effects) as compared to the lower rates. The black curves (named “x_fine” in the legend) represent values belonging to the finest mesh depicted in Figure 2(d). Their deformation patterns follow different trends compared to the rest of the curves, in particular, when the continuum damage model is used.

## 5. Concluding Remarks

One way of enhancing the implementation and computational efficiency of the simulation process is to use a decoupled formulation of the effective stress response from damage. Thereby, a common effective stress routine that serves as a base for the both failure models is used in present work.

A general conclusion from the simulation results presented in this paper is that a clear mesh dependency is present in all deformation modes and for all deformation rates, both for the uncoupled and for the coupled damage model considered in the investigation. The set of element sizes, deformation rates, and material parameters in the present investigation, relevant from the engineering point of view, appears not to yield any regularizing effect on the mesh dependency in the great majority of the cases (possibly all), despite the employment of the rate dependent damage models. As argued in the literature [16], one explanation is that, under the current “engineering” circumstances, the viscous regularization effect is too low. Furthermore, the effective viscoplastic regularization length decreases with the increasing damage (or deformation) values and when it reaches the characteristic element size, the regularization effect disappears. Consequently, a larger difference between the effective viscoplastic length scale and element size increases the regularization effect and could be accomplished by increase of viscosity, increase of the deformation rates, and possibly also further refinement of the mesh.

If the comparison is made between the continuum and uncoupled damage models in the shear test, a good agreement is obtained between the resulting force-displacement curves. The obtained differences between results based on the two types of damage models in terms of (in some cases) different crack patterns and energy needed to initiate the cracks, despite the adopted calibration procedure of the model parameters in order to obtain analogous behavior, possibly depend on the different nature of the damage models impact on the continuum behavior. In order to evaluate which failure model (and associated set of parameters) yields the most reliable results, physical experiments are needed. In general, it seems like, despite its restrictions concerning lack of damage influence on the continuum behavior, the uncoupled damage model gives more stable and realistic results, regarding crack development, compared to the present continuum damage model.

## Figures and Tables

**Table 1:** Material properties pertinent to elastic and thermal response.

*E* (GPa)	*ν*	*ρ* (kg/m^3^)	*T*_melt_ (K)
190	0.3	7850	1673

**Table 2:** Johnson-Cook parameters.

*A* (MPa)	*B* (MPa)	*C*	*n*	*m*	${\dot{\epsilon }}_{0}$
550	500	0.0804	0.4	1.68	0.001

**Table 3:** Calibrated uncoupled damage model parameters.

*d*_1_	*d*_2_	*d*_3_	*d*_4_	*d*_5_
0.26	0.614	2.557	−0.028	0.6
